# Operative Surgery

**Published:** 1851-08

**Authors:** 


Art. V.—*Operative Surgery.* By Frederic C. Skey, F.R.S. Philadelphia: Blanchard & Lea. 1851.

Mr. Skey has performed a highly acceptable service to the medical profession, by the publication of this admirable work. The author never attempts anything like extravagance in surgical art. His whole effort seems to be directed to the perfection of curative surgery, in order to leave as little for operative surgery to do as possible. We admire the tone of the following remarks, which we quote from Mr. Skey's preface:

"There is no department of medical practice more attractive to the younger members of our profession than that of operative surgery. Its brilliancy, its eclat, its critical influence on disease, all contribute to this popularity, and attach an interest to it in the minds of the student of medicine far beyond that connected with the study of any other department. The value of a hospital is appreciated by the number of its operations, not by the exhibition of the curative power of its surgical staff, and still less by the success which follows the frequent resort to the knife. The operating theater is a place of weekly resort, the 'high change' of the institution, at which we find united the largest assemblage of professors, of students, and their friends. Is not this enthusiasm somewhat misapplied? Between the claims to respect of curative and operative surgery, there is no comparison. I venture to say, with all respect to my many superiors in surgical acquirement, that it is the duty of the teacher to expatiate with all the power of his authority on the superiority of the one over the other.

"There is no greater obstacle to the advance of high classed surgery, than the man who, having acquired the reputation of a dextrous operator, regards the world as the great field of his experiments. A man who has the reputation of a 'splendid operator' is ever a just object of suspicion; and I firmly believe that the bad operator, conscious of his own weakness, will be found to have ever been the larger contributor to scientific surgical knowledge.

"There is a position which operative surgery is entitled to hold in our esteem, and which, so long as it does not clash with, nor interrupt the studious and persevering effort of the surgeon to render its aid necessary, must ever rank among the most valuable deparments of scientific medicine."

In another place Mr. Skey says: "I am old enough in my profession to have witnessed in past times much of wrong; I hope to live long enough to see this wrong converted into right. I have endeavored, as an English metropolitan surgeon, to carry into execution at least one primary object, viz: to strip the science of operative surgery of a false glare, mistaken by the ignorant for the brightness of real excellence, to check a spirit of reckless experiment, and to repress rather than encourage the resort to the knife as a remedial agent."

These are surgical sentiments that we admire, and we should be glad to see them sedulously cultivated. They are forcibly impressed by Mr. Skey in the various departments of his excellent work.

The first and second chapters of this work are devoted to what is usually denominated minor surgery, and are replete with valuable instruction.

The fifth chapter treats of the Bandage, and we are gratified in finding the opinions we advocated in a former volume of this Journal, abundantly and ably sustained by the high authority of Mr. Skey. On the subject of the bandage Mr. Skey says: "In no department of surgery is the improvement, which has of late years taken place, more strikingly exemplified than in this; and it is a fact well worthy of observation, that as we advance in scientific knowledge, so have elaborate and complicated contrivances gradually yielded to the simple bandage, now almost exclusively employed. This application, properly made, answers, with some few exceptions, every useful purpose, although skill and ingenuity may occasionally be required to meet the demands of individual cases.

"Bandages are applied for various surgical purposes, viz: to protect a diseased part from injury—to confine dressings, etc., in their situation—to restrain movement —to assist union where a solution of continuity has occurred—to support parts in a state of debility—to restrain hemorrhage by compression—to oppose certain displacements of the viscera, and to prevent or rectify deformity, by holding the dislocated parts in a natural relation to each other."

"Skill in the application of a bandage is not to be acquired by any description; it can only be obtained by frequent practice; and I would earnestly advise every student to lose no opportunity of becoming a thorough proficient in this department of his profession. It is a subject which, in relation to its importance, is neglected, I believe, beyond all others. A bandage is not successfully applied with the facility imagined by those unpracticed in the resort to it. Many surgeons, who can perform a capital operation, not without eclat, find great difficulty in the application of a simple bandage, and execute this really important part of their duty most imperfectly. Such an exhibition presents a warning and a useful lesson to all around. *There is no excuse for the neglect of this subsidiary art; the opportunities for practice are always abundant; there is every motive for the acquisition of skill, and its possession is a duty we are all liable to be called upon constantly to exhibit.*"

The chapter on Fractures is one of the very best on the subject that we have seen, although we do not approve the treatment in all respects. But there is much valuable instruction in this section, by which a surgeon who understands the correct treatment, may profit. These fracture cases are the sources of many of the suits for malpractice, a custom which is increasing rapidly, and which can only be arrested by a general elevation of the medical profession. The most skilful and competent are as liable to these suits as those who have scarcely a qualification for practice. But when the profession of medicine is a unit, and its members feel and show that he who is unworthy shall not disgrace their ranks, we may expect the profession, as a body, to speak authoritatively to courts of justice.

We have read Mr. Skey's remarks on aneurisms, varicose veins, amputations, excisions or resections, hernia, lithotomy and lithotrity, on the removal of cicatrices, and auplastic operations, on the Cæsarean section, and ovariotomy, and on operations upon the eye, with a great deal of satisfaction. Mr. Skey has done ample justice to most of the points involved in these subjects.

The chapter upon Lithotomy and Lithotrity contains a mass of interesting and valuable information, but we cannot agree with the author's views of the relative value of lithotomy and lithotrity. Nor do we believe that Mr. Skey himself would entertain these views if he were in full possession of complete statistics on lithotomy and lithotrity, and the processes by which Mr. Skey reaches his conclusions show how important statistical knowledge is in making up opinions. Mr. Skey nowhere refers to the invaluable contributions made to the statistics of lithotomy by Professor B. W. Dudley, of Kentucky, and any comparison between lithotomy and lithotrity in their results, that omits that contribution, must necessarily do injustice to lithotomy, and to surgical science. The unparallelled success of Prof. Dudley may be equalled by the use of the same skill, judgment, care, and prudence that conduced to his remarkable achievements. But we shall not dwell on this subject at present, as we shall shortly review the questions involved, in another field. We hold that surgical and medical practitioners cannot be too particular in their statements of scientific truths, because each item forms a portion of statistical knowledge that is to guide the future surgeon and physician, and for this reason, we recently called the attention of medical readers to the marvellous case of lithotrity reported by Professor Dugas, of Georgia. It is the most extraordinary case on record, neither Heurteloup, Civiale, nor any other lithotritist having recorded an achievement comparable to what Professor Dugas claims. We republished the paper of Professor Dugas in this Journal, and stated some of our difficulties in fully receiving all the statements made by the Professor. We were as respectful as possible, yet we understand that Dr. Ford, of Georgia, has made a bitter and offensive reply to our comments. We have not yet received the Georgia Journal, but as soon as we do so, we shall make a rigid examination of the extraordinary case. For the information of Dr. Ford, we must say that we have conversed with a number of lithotomists on Professor Dugas' case, and we have seen no one who did not take the same view of it that we did. But when we get to see Dr. Ford's response, we shall endeavor to elaborate our views upon lithotomy and lithotrity. We are full of faith in the superior merits of lithotomy, probably because we have seen its splendid triumphs for thirty years, in the hands of its greatest master, and we shall endeavor to show the grounds of our faith.

We take great pleasure in commending Mr. Skey's Operative Surgery to the favor of medical readers. A careful study of its chapters will prove to be its highest recommendation. As a guide in curative, as well as in operative surgery, we consider that the work has very high claims upon the attention of practitioners.

The mechanical and artistical departments of the work are highly creditable to Messrs. Blanchard & Lea. The typography is very beautiful, and the wood engravings are all that can be desired. B.

